# Woman-centered care 2.0: Bringing the concept into focus

**DOI:** 10.18332/ejm/91492

**Published:** 2018-05-30

**Authors:** Yvonne Fontein-Kuipers, Rosa de Groot, AnneLoes van Staa

**Affiliations:** 1 Rotterdam University of Applied Sciences - Research Centre Innovations in Care & School of Midwifery, Netherlands; 2 Sanquin Research, Netherlands; 3 Rotterdam University of Applied Sciences - Research Centre Innovations in Care, Netherlands

**Keywords:** woman-centered care, midwives, midwifery practice, advanced concept analysis, conceptual framework, care philosophy

## Abstract

**INTRODUCTION:**

Woman-centered care has become a midwifery concept with implied meaning. In this paper we aim to provide a clear conceptual foundation of woman-centered care for midwifery science and practice.

**METHODS:**

An advanced concept analysis was undertaken. At the outset, a systematic search of the literature was conducted in PubMed, OVID and EBSCO. This was followed by an assessment of maturity of the retrieved data. Principle-based evaluation was done to reveal epistemological, pragmatic, linguistic and logic principles, that attribute to the concept. Summative conclusions of each respective component and a detailed analysis of conceptual components (antecedents, attributes, outcomes, boundaries) resulted in a definition of woman-centered care.

**RESULTS:**

Eight studies were selected for analyses. In midwifery, woman-centered care has both a philosophical and a pragmatic meaning. There is strong emphasis on the woman-midwife relationship during the childbearing period. The concept demonstrates a dual and equal focus on physical parameters of pregnancy and birth, and on humanistic dimensions in an interpersonal context. The concept is epistemological, dynamic and multidimensional. The results reveal the concept’s boundaries and fluctuations regarding equity and control. The role of the unborn child is not incorporated in the concept.

**CONCLUSION:**

An in-depth understanding and a broad conceptual foundation of womancentered care has evolved. Now, the concept is ready for research and educational purposes as well as for practical utility.

## INTRODUCTION

Woman-centered care has been recognized as a marker of quality in maternity services^1^. This phenomenon has been derived from labels such as person-, patient- or clientcentered care. These concepts relate to various healthcare contexts^1-4^. The concept label woman-centered care is used in relation to midwifery care because woman-centered care emphasizes a strong midwifery-specific focus^5^. Woman-centered care has been acknowledged in policy and organizational documents^5-12^. Woman-centered care prioritizes the woman’s individual unique needs, as defined by the woman herself — assigning to the woman choice, control and continuity of care^5-12^. This concept description has resulted from consultation with women (recipients of maternity services), midwives, obstetricians and policy makers^10,13,14^ but lacks a scientific theoretical foundation.

Woman-centered care has become more widespread and elements of the concept have been addressed in diverse research contexts (e.g. shared decision making, continuity)^15-19^. It has been debated whether womancentered care is suitable solely for the healthy childbearing woman or may also enhance the experience for the woman with health and/or psychosocial problems^1^. Unclear use of woman-centered language may affect correct understanding of the concept^20^. This may result in different interpretations of woman-centered care, requiring clarification of the concept^21,22^. An earlier concept analysis of woman-centered care^23^, that was criticized for not presenting a scientific description of the concept^24^ was based on nursing studies whereas midwives are regarded as the main professionals caring for the childbearing woman^25^.

Given the impact of the concept, a research team was formed to further investigate the concept. It was felt that the concept woman-centered care is affected by conceptual evolution, demanding a greater precision in meaning and advancement towards greater utility in midwifery theory and practice. Therefore, a thorough re-analysis of the concept from a midwifery-science perspective is justified.

In this paper, we present a theoretical analysis of the concept ‘woman-centered care’ as it appears in scientific studies in the midwifery domain, using the steps of concept advancement^26-28^. We aimed to ensure that our analysis targeted practical relevant ideas and therefore based it on actual uses by midwives. The aim was to analyze closely the central meaning and the core components of the concept. The outcome of this study will be a theoretically-based definition that incorporates understanding of woman-centered care for midwives.

## METHODS

The first step of concept advancement is to collect and assess appropriateness and maturity of retrieved data. The second step is to re-examine the data, using four principles representing the major perspectives of the philosophy of science: epistemological, pragmatic, linguistic and logic — a so called principle-based evaluation^27^. In order to advance the concept, the third step involves formulation of key questions for which answers are derived from the data. Findings of these processes are then integrated in a final step — the theoretical definition^29^.

### Search strategy and study selection

A search strategy and audit trail to systematically collect data explicitly focusing on woman-centered care was undertaken. To ensure a high degree of subject specificity^30^, data were based on literature of midwifery, healthcare, healthcare education and social sciences, as these sources can contribute to a unique perspective of the understanding of woman-centered care. We included original, peer-reviewed research with participants of all ethnicities, performed in any country.

Two authors [YF, RdG] independently searched the literature in the electronic databases PubMed, OVID and EBSCO (1 September – 30 June 2017) using the following search terms: [woman OR women OR client OR patient] AND [centred OR centered OR focused] AND [midwife OR midwives OR midwifery care] AND [practice OR experience OR view]. Preliminary literature searches showed us that these terms were most suitable as conceptual key terms. We placed no limits on the publication date, as centeredness in healthcare is not a contemporary concept^31^. All full text articles published in English were considered eligible.

**Figure 1:**
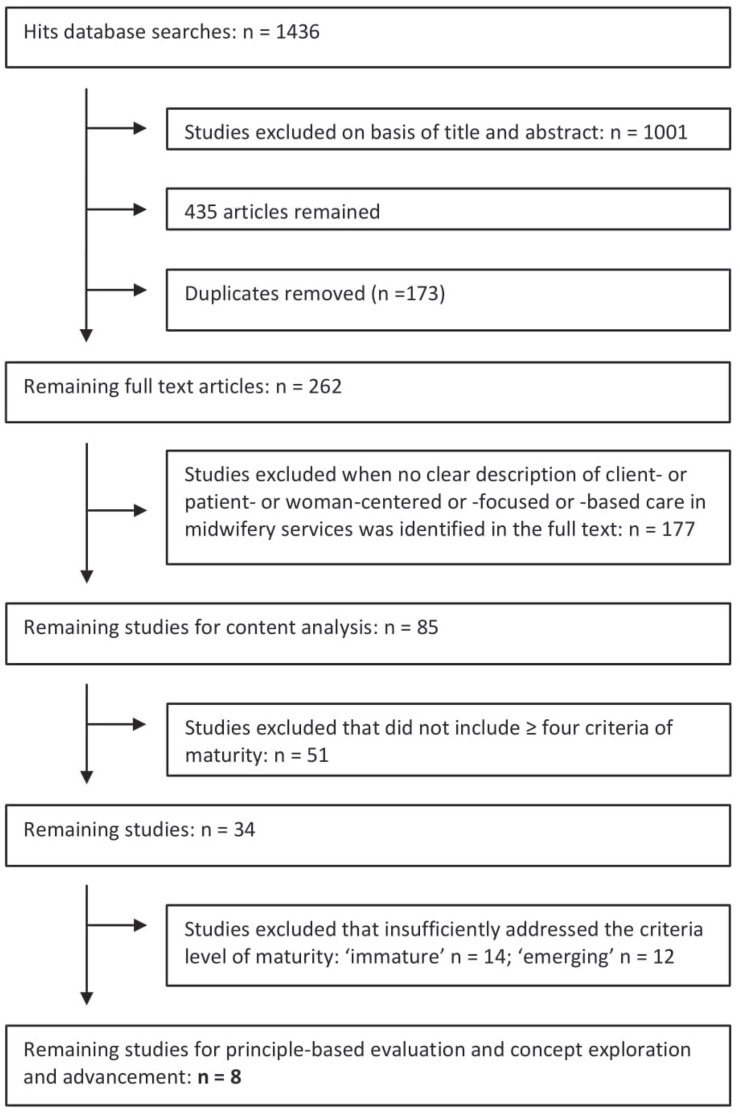
Flow chart

The initial search identified over 1400 research entries. After scanning titles and abstracts for a clear relevance to woman-centered care or an equivalent synonym (i.e. patient, client, person) and removal of the duplicates, the selection was narrowed down to 262 articles that were scrutinized in full text. The remaining papers either aimed to develop a woman-centered care model, studied the utility of womancentered care in midwifery practice or sought understanding of the concept. Relevance to the subject was then judged by assessing if a clear description of woman-centered or its equivalent (client-centered; patient-centered; personcentered) was identified in the context of midwifery services, after which 85 articles remained (Fig. 1).

### Assessment of concept maturity and study selection for principle-based evaluation

A second selection constituted a purposive sampling for content analysis of the remaining 85 full text articles, based on *a priori* criteria that at least four of the following five criteria had to be discussed in the study: 1) definition of womancentered care or its equivalent, 2) attributes, 3) antecedents, 4) outcomes, and 5) boundaries. These criteria define the level of maturity of the concept studied in each individual paper^28,32^ (Table 1). At this stage of the process, in an effort to seek various perspectives, studies using qualitative and quantitative data were included^27,33^. The articles did not include any conceptual or theoretical papers as these did not appear in our full text selection.

**Table 1:** Included studies and criteria of maturity for analysis

	*Aim study*	*Study design*	*Sample*	*Definition*
***Study 1***	To develop a model of woman-centered care^39^	Mixed-methods design: Hermeneutic design - 12 articles followed by 6 focus groups discussing the themes that emerged from the literature	Swedish practicing midwives (n = 30). Age 28–64 years, 1.5–43 years’ work experience. Practicing in primary care (home birth and birth centers) secondary and tertiary hospital (labor ward) settings	A balancing act of creating reciprocal relationship and a birthing atmosphere, and using grounded knowledge to provide care based on a midwifery model of care while handling the hindering cultural norms
***Study 2***	To develop a conceptual model for midwifery practice41	Qualitative; unstructured interviews- Grounded Theory	Midwives n=250 (New Zealand 137/ Scotland 113) - independent community based and hospital-based midwives. Women n=218 (New Zealand 109/ Scotland 110)	A relationship between a woman and the midwife centered in 'being' rather than 'doing’, which is episodic and not always equally balanced
***Study 3***	To propose a model how power between women and midwives can be shared in midwifery practice^37^	Mixed-methods design: Crosssectional study - triangulation of survey and interviews	Interviews: New Zealand team and hospital-based midwives (n=11). Survey: New Zealand women (n=72) from different ethnic groups. Mean age 22 (15–35) years. Midwives (n=41) originating from New Zealand, Australia. The majority (35/ 85%) trained as registered general nurses prior to their midwifery education and six were direct entrants. Over half (22/54%) had three plus years delivery suite experience, 12/29% had less than oneyear experience and 7/17% had one to three years. 18/44% were working in independent practice, 16/39% were hospital based and 7/17% worked in a domino team scheme	Working together towards a common aim while women and midwives can define their individual and joint accountabilities as well as their ethical responsibilities to each other, whilst sharing the decision making
***Study 4***	To examine the role of midwives from the perspectives of women and midwives to identify key elements to be required of a midwife^38^	Mixed-methods design: surveys and interviews	Australian midwives and women from various states and territories. Survey: Women (n=28) from metropolitan, rural and remote areas, and hospital and birth center birth experiences. Midwives (n=80). Interviews: 32 (independent) practicing midwives and practicing nurses with midwifery qualifications.	To work in an enabling or ‘empowering’ way, including working in respectful partnerships with women to ensure that they develop confidence and are able to make decisions for themselves and respecting women’s time, their families, their fears, their choices and their need for information
***Study 5***	To explore how caseloading midwives construct midwifery^42^	Semi-structured interviews	Caseload midwives (n=48) practicing throughout New Zealand	Care with a safe outcome for mother and infant, where it is the woman's experience rather the outcomes that are central
***Study 6***	To offer insights into the experiences of midwives about continuity of care^40^	Mixed-methods design: Qualitative descriptive research followed by interviews exploring gaps in the literature	New Zealand caseload midwives (n=11) from rural and urban areas with 8 to 20 years work experience.	Midwifery care philosophically based on the woman-midwife relationship being one of partnership, reciprocity and trust
***Study 7***	To explore women’s understanding of midwives and maternity care^43^	Mixed-methods design: Qualitative descriptive research followed by interviews exploring gaps in the literature	Young New Zealand females (n=11) who had (not) attended a birth or were mothers	Client/midwife relationship based on shared power and familiarity, including a supportive midwife, prepared to respect both the philosophical beliefs and clinical preferences of the client
***Study 8***	To seek understanding of how woman-centered care was interpreted and experienced in practice^36^	Mixed-methods design: National postal survey followed by interviews	Midwives and supervisors* in from three UK maternity units from various locations, size and type. Survey: Practicing midwives in DomInO care, caseload, community care, teammidwifery and home birth (n=771) and coordinating supervisors of midwives*. Interviews: Midwives (n=90), midwifery supervisors*, managers*, doctors* and lecturers* and mothers (n=115)	The needs of the woman at the heart of healthcare
	***Attributes***	***Antecedents***	***Outcomes***	***Boundaries***
***Study 1***	Reciprocity Creating trust and safety	Embracing a woman-centered philosophy of care Increase in interventions	Strengthening the individual woman’s own resources and her sense of coherence Normality of childbirth Choices for women Humanization of childbirth	Safety Medical treatment Structure healthcare organization Work culture
***Study 2***	Acknowledging the woman as the expert of her life and environment Continuity of care Joint informed choice and decision making Reciprocity Established relationship	Renaissance of midwifery	Authenticity of midwifery/differentiation from other disciplines in maternity care services	Discrepancies between midwife’s and women’s aims and aspirations
***Study 3***	Informed decision-making Joint decision-making Teamwork between woman and midwife Establishing a relationship	Women voicing their needs regarding maternity services Women participating in activities of professional organizations	Inequality in the midwifewoman relationship Quality of care	Health risks Professional disciplinary power
***Study 4***	Continuity of caregiver Discussing options, expectations and realities Collaboration with other healthcare providers Being available Creating trust Practice based on evidence	Changing role of the midwife and the need for national competency standards Articulating future direction of the midwifery profession Need of philosophical underpinnings to practice	Challenging practice A clear articulation and understanding of the role of the midwife	Structure healthcare organization Medical dominance Midwives’ family responsibilities and social commitments Resources (i.e. finances)
***Study 5***	Continuity of care	Embracing a womancentered philosophy of care Women/ childbirth activists/ consumers voicing their needs regarding maternity services Evaluation of care (model) Midwifery practice partners are philosophically aligned Fundamental commitment to making a difference at an individual, community and societal level	Changing healthcare context with midwife as lead-carer Reduction of interventions/de-medicalization	Medical approach Safety Political strategies/ agenda
***Study 6***	Continuity of care Autonomy of the woman	Evaluation of care (model) Midwifery practice partners are philosophically aligned Fundamental commitment to making a difference at an individual, community and societal level	Joy in work/ midwifery practice	Not mentioned
***Study 7***	Continuity of carer throughout childbirth process Development of deep familiarity through assessment Education Informed decision-making Being the woman’s advocate Being unequivocally available	Midwives’ needs to express their midwifery profession due to loss of credibility (media)	Trust in midwifery care	Health risks Unachievable and unsustainable expectations that women place on midwives Women’s demand for nonnegotiable care
***Study 8***	Partnership between woman and midwife Caseload midwifery Facilitating choice	Governmental evaluation of maternity healthcare system Recognition of women’s rights in childbirth Need for humanization of childbirth Midwives’ convergence about the actual philosophy of woman-centered care	Reduction intervention rates Input for midwifery education	Lack of resources (i.e. staff, time) Safety (Local) policies

* Participants not included in analysis

Two researchers [YF, RdG] independently applied the criteria. MS Excel was used to assist with organizing and analyzing the data according to the criteria and to compare input. A selection of 34 eligible papers emerged. Selection showed a substantial Kappa of 0.64 between the researchers^34^. We discussed the level of maturity of the concept’s content criteria described in each individual paper, categorizing this as ‘immature’ (criteria inadequate, confusing and/or competing), ‘emerging’ (criteria partially operationalized) and ‘mature’ (criteria theoretically used and operationalized)^32^ (Fig. 1). Consensus was reached on a final selection of eight studies that showed clear presence of concept maturity (Table 1). The 26 papers excluded had one or more criteria with an insufficient level of maturity, predominantly concerning: definition, antecedents and outcomes. It was concluded that the remaining papers were theoretically rich. The texts of the included papers served as data for our principle-based evaluation^27-29,32^.

### Concept exploration and advancement

In the process of concept advancement it is assumed that unanswered questions remain^28,32^. In order to advance the concept, critical and analytical questions that reflected issues that emerged from the data were postulated and discussed with the authors, to ensure relevance, logic and comprehensiveness (Table 2). The texts of the eight selected articles were independently examined by two authors [YF, RdG] to answer the key questions that derived from these texts^28,32^. Answers were discussed among all authors and developed as concept components^28,35^.

**Table 2:** Concept exploration and advancement

*CRITICAL QUESTIONS*	*ANSWERS ABSTRACTED FROM THE DATA (8 studies)*
***Q1. Who or what is the focus of woman-centered care?***	Woman-centered care is a process of partnership, interdependence, interconnectedness and co-creation neither the woman or the midwife is at the center of care^39-41,43^.
***Q2. Who has the locus of control in woman-centered care?***	Authoritative knowledge, perceived authority, medical procedures and expertise of the midwife can create imbalance in woman-centered care. Non-assertiveness and compliance of women and when women perceive the midwife as more knowledgeable, affect the collaborative relationship and shifts the locus of control^36,37,41,42^. The woman’s experience of feeling in control depends on who perceives to be in control of the care process and how both the woman and midwife perceive their respective role in the relationship^39,40,43^. When complications arise during birth, women are more inclined to hold the midwife accountable for the safety of the baby, due to perceived locus of control and authoritative knowledge^38,43^.
***Q3. What is the function of the relationship between the woman and the midwife in woman-centered care?***	The relationship serves as the opportunity for cooperation between woman and midwife; to enable tailored care, to develop trust in order for the woman to feel in control by actively giving the power of decision to a person the woman trusts to make decisions in her advocacy and with grounded knowledge^36-43^. The relationship offers the opportunity to establish the boundaries for both parties and to recognize other constraints that may impinge on their responsibilities^37,39,40^.
***Q4. Is an equal balance between the woman and the midwife realistic in womancentered care and who or what defines the autonomy in setting boundaries of woman-centered care?***	The relationship between the woman and midwife in woman-centered care is grounded in equal humanity^37,40^. Woman-centered care provision, has a rather flexible, episodic and dynamic nature^36,38,40^, depending on individual needs, knowledge, expertise and values of the care provider (midwife)^36,37,39-41^, the receiver (woman)^37,39,43^ and the organizational flexibility in the healthcare system^36-39^. Reciprocity shifts together with the risk level concerned with the decisions at hand^36-40,43^, where women accept professional knowledge and authority of the midwife^37,38,43^. Midwives allow women to make choices^36^.
***Q5. What is the position of the unborn child in womancentered care?***	The (unborn) child was mentioned as a facilitator of transforming the woman a mother^36,39,40^.
***Q6. Are all characteristics of woman-centered care essential to provide ‘true’ woman-centered care?***	Commitment of midwives to the philosophy and woman-centered care model and involvement with women on practice, personal and emotional level^37,39-41^. The midwife intrinsically perceives woman-centered care as true midwifery care^40,42^.
***Q7. Who is the (true) partner in woman-centered care?***	The midwife is the woman’s most prevalent partner^36,37,39,41,42^. Women should be encouraged to seek their own relationships in order to share the responsibility to support the woman’s choices with confidence and knowledge by significant others^38^.
***Q8. Do midwives work ‘for’ or ‘with’ women in woman-centered care?***	The midwife is the woman’s partner^37,38,40^ and supporter^39-41,43^. The midwife is a mediator^38,42^, a facilitator^36^, and a coordinator^42^.
***Q9. What is the ultimate consequence of a lack of woman-centered care?***	Unsafe and unnecessary interventions^39,42,43^. Affected women’s human rights^36^. The loss of the midwife’s autonomy and identity^37,41,42^. Loss of the physiological approach of childbirth39.
***Q10. How can woman-centered care be measured for research purposes?***	Continuity of care^25,27-29^, continuity of carer^36,38,43^, and accessibility of the midwife^38,43^. Pregnancy and birth outcomes^39^. (Women’s) experiences of the woman-midwife relationship and partnership^36,37,39,41^, reciprocity^39,41^, joint decision-making^36,37,41,43^, and (women’s) experiences of safety and trust^38,39^.

## RESULTS

### Sample characteristics

The final sample of eight studies (Table 1) included those that were published between 1998 and 2014, of which 75% were published in the last ten years. Five studies used a mixed-methods approach with triangulation of data from questionnaires and interviews^36-38^, or collected data from literature and focus groups^39,40^. Three studies applied a qualitative design including individual interviews^41,42^ or focus groups^43^. The studies originated primarily from New Zealand^37,40-43^, Australia^38^, Sweden^39^ and United Kingdom^36,41^. A total of 1364 midwives and 444 users of maternity services were included. All but one study addressed all five criteria of concept maturity.

Studies contained midwives practicing in hospital-based care settings^36,37,39,41^, in primary care^36,38,39,41^ and homebirth^36,38,39^. Midwives were either independent practitioners^38,41^ or were employed; practicing either as an individual^36,40,42^ or as a member of a team of midwives^36,38^. Four studies included women that had experience with midwifery care^38,39,41,42^ or with women requiring preconception services^43^.

### Principle-based evaluation of woman-centered care

The epistemological principle guided an examination of how clearly woman-centered care has been defined in the literature. The pragmatic principle guided the examination of the concept’s usefulness for midwifery. The linguistic principle guided an in-depth evaluation of the consistency of use and meaning of the concept, and the logical principle guided a precise examination of the concept’s systematic interrelationships with other concepts without losing its boundaries^27-29,32^. We summarized key elements of the data (e.g. complete citations, segments of text bearing implied meaning) in tabular format using MS Excel. To enhance the validity of each entry, we reviewed original data and entries to ensure that the table was comprehensive. Forty-eight, randomly recruited, practicing midwives served as content experts and were individually challenged by the authors to cite appropriate evidence in support of the analysis. Their answers were used to validate the findings found in the literature with a focus on their relevance for midwifery practice in order to maximize the value of our results. The midwives shared their actual experiences of woman-centered care, agreed with the content and the relevance of the epistemological principles and directed us to literature that we used in writing the discussion of this paper. This collaborative analysis enhanced the credibility of the findings. The summative conclusions (i.e. findings of the four guiding principles) are presented.

#### Evaluation of the concept’s definition and differentiation from other concepts

The concept was not defined explicitly in any of the retrieved literature but implicit meaning abounded. The common denominator in the data was the dual womanmidwife relationship characterized by dynamic interaction between the woman and the midwife^37-41,43^. The emphasis was on relational care, implying an active liaison process between the woman and midwife. There were examples indicating that women and midwives share a common aim with individual and joint accountabilities^36,37,42^. For example, a safe outcome is such a joint goal between the woman and midwife^36,42^. The woman herself is accountable for her own health behavior^37^ or for explicating her own needs and values^37,38,42,43^. The midwife’s responsibility is to be (continuously) available^38,43^.

In woman-centered care there is no prioritizing of safety versus the woman’s experience^36,42^. Woman-centered care does not only encompass the physical parameters of pregnancy and birth but includes, with similar importance and focus, the woman’s psychosocial dimensions — these are mutually constitutive^36,42^. There is no subjectiveobjective tension. This implies that woman-centered care differs from the biomedical and biostatic concepts of health^4,44^. In our studies, there was no emphasis on the formal client side of the relationship. Referring to the woman as client or customer suggests a financial reimbursement and an agreement between the woman and the midwife with a business or commercial interest. This demonstrates that woman-centered care is distinct from healthcare business^45^. Throughout the literature there is strong evidence that boundaries of woman-centered care were primarily experienced by the application of guidelines and policies^36,37,39,42,43^, demonstrating that protocol-based care delimits the concept^46^.

#### Evaluation of the concept’s applicability and its usefulness to the discipline

When considering the range and depth of applications of woman-centered care, it’s utility appears quite high. Woman-centered care is structured in the caseload model of care^42^, the partnership model^37,40,43^ and the midwife-led care model^36,38,39,43^. Utilized woman-centered strategies are continuity of care, one-to-one care and continuity of care-giver^38,40-43^. Implied meaning of these strategies is that the concept is regarded as both a task and as a system^15^ through evidence of relational continuity^36-38,43^, management continuity^40-42^ and continuity of information^37,41,43^. The strategies facilitate tailored care, shared-decision making, and the autonomy of the woman during the childbirth process^37,40-42^. Not surprisingly, collaboration and communication are required midwives’ competencies^37-39,42^.

It became apparent that evaluation or criticism of existing midwifery services^38-40,42^ always precedes the implementation and utilization of woman-centered midwifery care^36-43^. This implies that providing woman-centered care is a conscious act of or a deliberate change in care conceptualization and provision.

#### Evaluation of consistent and appropriate use of the concept in context

Some studies referred to woman-centered care as a philosophy (i.e. an abstract perspective)^37,40-43^ while others regarded it as a midwifery framework or tool (i.e. a pragmatic perspective)^36-39,41^. This could cause confusion concerning the level of abstractness of the concept. It seems that both viewpoints are accepted in midwifery, although the philosophy needs to precede practical utility^38,41,42^ — thus being a precondition. Commitment to the philosophy and sharing the philosophy of woman-centered care among midwives facilitates its practical application in care provision^36,40^ and can also be regarded as a prerequisite.

Linguistic properties of the relationship in womancentered care are reflected by terminology such as balancing act and reciprocity^39,41^. Hence, words such as *joint, shared and mutual* ^37,40,41^ reflect the dual relationship in the woman-midwife relationship. These words suggest an on-going equity between the woman and the midwife, although the relationship is rather episodic in nature^41^. In woman-centered care, the woman is regarded as the expert of her own life and body, while the midwife is the professional expert of pregnancy and childbirth^37,41^, implying that the input and interactivity of both the woman and the midwife depend on context and situation where the woman’s experiential knowledge is considered legitimate. The phrases *autonomy* ^40,41^ of the woman and a nonauthoritarian approach of the midwife^39^ outline the focus on the woman. The words established^37,41^ and availability^39,40^ imply the quantity and intensity of (time) investment in the woman-midwife relationship and the engagement between both actors. The broad scope of current applications reinforces the fact that the concept denotes a relatively dynamic process.

#### The concept’s boundaries when integrated with or related to other concepts

Midwives in the selected studies valued the importance of maternal and neonatal health outcomes, suggesting some overlap with patient-centered care^4,47^. Despite the acceptance of technical attributes^36,39^, the guiding principle in woman-centered care is appointed as the physiology of pregnancy and birth^36,37,39,42,43^. Hence, referring to the woman as a patient^36^ implies the existence of ill health, while womancentered care frames health through the lens of wellbeing by means of supporting and enhancing the physiological process of childbirth^39^. Therefore, woman-centered care fits in a biopsychosocial model of childbirth rather than in a biomedical model of care^48,49^.

Woman-centered care includes supporting the woman’s autonomy, respecting her values and engaging the woman in her care process^39-41^. This shows an interrelationship with value-based care^50^, self-management^51^ and customerorientated care concepts^52^. Humanism is frequently mentioned in the context of woman-centered care^36,38^-^40,42,43^ and implies linkage to a woman’s sense of coherence, salutogenesis^53,54^, and with holism^55^ — all associated with the life-world orientated care model^55^. Womancentered care considers the woman’s processes and experiences^37-39,41-43^, showing an overlap with the life-world approach as used in social and behavioral sciences^56,57^. In summary, woman-centered care seems to be part of a continuum when positioned theoretically with other concepts and is more deeply epistemological in emphasis than currently presented in the professional arena. The concept is multi-dimensional, qualitative, contextual and rather complex.

### Concept advancement: using the literature as data

As expected, specific analytical questions emerged during the process of data analysis^28,32^, which we formulated in ten critical questions (Table 2). We returned to the selected eight studies, executing these critical enquiries to determine congruence among perspectives and to reveal the conceptual components: antecedents, attributes, outcomes and boundaries^28,35^.

#### Antecedents of woman-centered care

Providing woman-centered care is a conscious act and deliberate choice, instead of a care concept being applied by default. A process of (transformational) thinking and reflection on willingness regarding the commitment to the philosophy are recognized prerequisites for providing woman-centred care.

#### Attributes of woman-centered care

The term woman-centered care suggests that the childbearing woman is at the center of care. ‘Care’ has an active and a passive form, you can either care for someone, or you can be cared for by someone else. This would make the midwife the active and the woman and child the passive recipients. According to our studies, the focus of womancentered care does not define the relationship in this way, but rather stresses the dynamic and reciprocal character of the woman-midwife relationship. Semantically, it can be questioned if ‘care’ is correctly fitting the meaning of the concept. Our studies highlighted that the midwife’s role as communicator, collaborator and leader^58^ is truly essential for providing woman-centered care.

#### Outcomes of woman-centered care

The positive outcomes of woman-centered care when present, as shown in Table 1, imply that there might be consequences when woman-centered care is lacking, including dehumanization and depersonalization of care, often coinciding with medicalization and a loss of quality of care. Therefore, the positive and negative outcomes require monitoring. The studies suggested that there are various parameters of woman-centered care that can be measured in quantitative research. Subjective aspects are more eligible for qualitative research methods.

#### Boundaries

One of the key features of woman-centered care is partnership, however, this is not infinite. The boundaries of the partnership are defined by the elements control and equity. Although all our studies emphasize the importance of the woman feeling in control, ultimately it is both the woman’s and midwife’s sense of own autonomy that influence the extend of being in control and having control. Boundaries of control thus shift. Partnership suggests a static model of equal balance that is true for every individual and in every situation or context. Equality here, however, seems to be a continuum where the boundaries are set by the professional capacity of the midwife, the woman’s experiential knowledge and assertiveness, compromised health and the healthcare system. These bounding elements determine why womancentered care differs from the partnership model^59^. In addition, the boundaries of woman-centered care are also set by individuality of the midwife’s intrinsic value of womancentered care and to what extend the midwife shapes it in daily practice.

Woman-centered care seems to be bounded to the woman-midwife relationship. The strong emphasis on this relationship during the childbirth process suggests limiting the woman’s relationships with her significant others during this period. Although it has been recommended to place the mother and the child at the center of care^6,10^, in our studies there was no explicit referral to the role of the fetus or neonate; only as an imperative to make the transition to motherhood feasible. This reinstates the woman as the central agent in childbirth rather than the child. Use of the term woman-centered care creates a boundary as it only refers to the woman and not to (the relationship with) others.

#### Definition

As a result of the findings of both the principle-based evaluation and further concept exploration and advancement, a definition (Box 1) of woman-centered care could be formulated, incorporating the meaning of the concept for midwives, utility, context, antecedents, attributes, outcomes and boundaries of the concept woman-centered care.

**Box 1. Definition of woman-centered care**

Woman-centered care is a philosophy and a consciously chosen tool for the care management of the childbearing woman, where the collaborative relationship between the woman - as an individual human being - and the midwife - as an individual and professional - is shaped through cohumanity and interaction; recognizing and respecting one another’s respective fields of expertise. Woman-centered care has a dual and equal focus on the woman’s individual experience, meaning and manageability of childbearing and childbirth, as well as on health and wellbeing of mother and child. Woman-centered care has a reciprocal character but fluctuates in equality and locus of control.

## DISCUSSION

By means of a theoretical analysis, we have gained a more in-depth understanding of the concept of woman-centered care, by creating a conceptual foundation, formulating a definition — specifically for the midwifery domain. To our knowledge the concept has not been approached in such a manner before. The strength of this method is the summative conclusions according to the perspectives of the philosophy of science combined with the integration of the conceptual components that attribute to woman-centred care, as reflected in scientific literature^29^.

Woman-centered care places equal emphasis on women’s experiences and on clinical outcomes. Focusing on the value of positive experiences rather than purely clinical outcomes is consistent with the principle that a woman is not only a means of production and that midwifery care should therefore also focus on the subjective and experiential elements of childbearing, and on intended meaning of pregnancy and birth^60^. However, we don’t know exactly to what extent women consider their experiences to be of value compared to the opinions of maternity practitioners — or are able to stand their ground when their personal experiences are at stake. It might be that women are yet not used to sharing these thought processes as a result of traditional provider-driven care.

A noteworthy finding is the limited attention in our studies for the (unborn) child. While clinical outcomes are part of the midwife’s focus in woman-centered care, it can be assumed that when caring for a pregnant or birthing woman, the midwife includes the health and wellbeing of the fetus in her care provision^6^ — although this was not explicated as such. In our analysis, woman-centered care was identified as a philosophy. From a philosophical perspective, focus on the woman is most clearly articulated in Kantian ethics: treat a person as an end and never simply as means to the end of others (Kant – Metaphysics of moral)^61^. This ethical statement seems to be reflected in our findings.

When midwives adhere to the biopsychosocial model of care instead of a biomedical model of care, this may contribute to the explanation why sometimes tension between midwife and obstetrician regarding management of care arises; caused by a paradigm disparity^49,62^. In order to adopt or implement woman-centered care, a position statement about the value of woman-centered care supported by both maternity care provider groups, policy makers, as well as healthcare insurance companies, would be constructive^21,22^.

We identified several ultimate outcomes of a lack or limited woman-centered care provision^36,37,39,40,42,43^ (Table 1). Women sometimes choose to ‘freebirth’ as a result of poor experiences with maternity care, a lack of faith in, and feeling unsafe with, the care provided — due to negative and disrespectful interactions with healthcare providers^63^. Woman-centered care is strongly associated with human dignity. It can therefore be suggested that an ultimate consequence of lack of woman-centered care includes non-attendance or can potentially result in legal claims recognizing that poorly experienced care is a violation of human dignity^64,65^. Midwives can also experience certain consequences when woman-centered care is lacking. Recent evidence suggests that midwives leave the profession due to not having more time to spend on giving women and their families high quality care. This influences their motivation to provide maternity care that does not coincide with their perceptions of how to offer authentic midwifery care to women, i.e. woman-centered care^66^.

In our selection of papers there was scant referral to the woman’s partner and/or the (unborn) child. This might suggest this is seemingly an unexplored aspect, requiring further attention and explication. Family-centered care^68^ might serve as a source of inspiration as how to address this issue. Real woman-centered care should look at how to include and consider the other significant parties. Attention to these issues could be part of (lifelong) education, research or serve as a topic for supervision.

Our definition acknowledges the humanistic nature of the woman-midwife relationship and enhances certain aspects: founding midwifery care with an underlying philosophy; the dual and equal focus of the concept; and the character and dimension of the relationship, acknowledging the humanistic nature — recognizing these as playing a key role in the woman-centered care concept. Our findings suggest that the term woman-centered care causes practical limitations, as the main focus is on the relationship; instead of the woman being at the center of care or having ultimate control. It could therefore be debated whether woman-centered care is the correct term. Based on our findings, relationshipbased personalized care might be more appropriate. When approaching our findings from a social perspective, womancentered care seems to be related to midwives’ behavior, being largely influenced by attitude and social influences^69^. We concluded that woman-centered care is not routine practice or care behavior per se. This would require a structural shift, based on more research and the implementation of the philosophy and the pragmatic and behavioral aspects of woman-centered care into midwifery education and practice. In order to adopt woman-centered care as ‘business-asusual’, requires fundamental changes in midwives’ attitudes; beliefs and willingness to provide woman-centered care are yet to be overturned^21^. These aspects might serve as parameters for future research.

Our study showed some limitations. The fact that we established woman-centered care as a rather mature concept was based on studies predominantly from countries^36,37,40-42^ that recognize woman-centered care as a standard way of practice^67^. The established level of maturity in our study might therefore not be generalizable to midwives in other countries, other than those of the included studies. It might however, provide valuable information for midwives in countries where woman-centered care is not established practice yet. The rather descriptive nature of our criteria for establishing the level of maturity of the studies might have been subject to selection bias of the two authors, although there was substantial inter-rater reliability. Also, we included a small sample of studies, which could have introduced overgeneralization of the analytic findings^27^. Still, we believe that the data set accurately represents the current state of science. The original studies used different methodologies with different levels of evidence. This can be regarded as a limitation of the study. However, we believe that including different methodologies could have contributed to a deeper understanding of the concept. Although the body of literature was adequate, it included predominantly research with a midwife focus – this being consistent with the scope of the study. We did not include findings of other healthcare professionals, although in reality they are present in integral maternity services. This indicates that our current use of the concept womancentered care builds on a fairly narrow understanding of the term woman-centered — however, it fits in with the midwife’s practice. A future across-discipline analysis can be recommended to reveal insight with potential for integration of varied disciplinary perspectives and crossboundary components.

## CONCLUSION

The aim of this advanced concept analysis was to secure practical relevance of what is mainly a theoretical, analytical exercise instead of a systematic review. We have simply systematically and methodologically consulted a sample of literature to make sure we have analyzed and synthesized the concept and its features in actual use among midwives, resulting in an in-depth understanding and theoretical conceptual foundation of woman-centered care. Now, the concept as presented, provides food for thought for researchers and educators, as well as for the realization of coherent policies and practice, on macro, meso and micro level.
